# Silver(I) nitrate two-dimensional coordination polymers of two new pyrazine­thio­phane ligands: 5,7-di­hydro-1*H*,3*H*-dithieno[3,4-*b*:3′,4′-*e*]pyrazine and 3,4,8,10,11,13-hexa­hydro-1*H*,6*H*-bis­([1,4]di­thio­cino)[6,7-*b*:6′,7′-*e*]pyrazine

**DOI:** 10.1107/S205698902000362X

**Published:** 2020-03-13

**Authors:** Tokouré Assoumatine, Helen Stoeckli-Evans

**Affiliations:** a Institute of Chemistry, University of Neuchâtel, Av. de Bellevax 51, CH-2000 Neuchâtel, Switzerland; b Institute of Physics, University of Neuchâtel, rue Emile-Argand 11, CH-2000 Neuchâtel, Switzerland

**Keywords:** crystal structure, thio-substituted pyrazines, pyrazine­thio­phanes, *ortho-*bis-*L* regioisomer, silver(I), nitrate, two-dimensional coordination polymers, supra­molecular frameworks

## Abstract

The two new pyrazine­ophanes, 5,7-di­hydro-1*H*,3*H*-dithieno[3,4-*b*:3′,4′-*e*]pyrazine, C_8_H_8_N_2_S_2_, **L1**, and 3,4,8,10,11,13-hexa­hydro-1*H*,6*H*-bis­([1,4]di­thio­cino)[6,7-*b*:6′,7′-*e*]pyrazine, C_12_H_16_N_2_S_4_, **L2**, both crystallize with half a mol­ecule in the asymmetric unit; the whole mol­ecules are generated by inversion symmetry. The mol­ecule of **L1**, which is planar (r.m.s. deviation = 0.008 Å), consists of two sulfur atoms linked by a rigid tetra-2,3,5,6-methyl­ene­pyrazine unit, forming planar five-membered rings. The mol­ecule of **L2** is step-shaped and consists of two S–CH_2_–CH_2_–S chains linked by the central rigid tetra-2,3,5,6-methyl­ene­pyrazine unit, forming eight-membered rings that have twist-boat-chair con­fig­urations. In the crystals of both compounds, there are no significant inter­molecular inter­actions present. The reaction of **L1** with silver nitrate leads to the formation of a two-dimensional coordination polymer, poly[(μ-5,7-di­hydro-1*H*,3*H*-dithieno[3,4-*b*;3′,4′-*e*]pyrazine-κ^2^ *S*:*S*′)(μ-nitrato-κ^2^ *O*:*O*′)silver(I)], [Ag(NO_3_)(C_8_H_8_N_2_S_2_)]_*n*_, (**I**), with the nitrato anion bridging two equivalent silver atoms. The central pyrazine ring is situated about an inversion center and the silver atom lies on a twofold rotation axis that bis­ects the nitrato anion. The silver atom has a fourfold AgO_2_S_2_ coordination sphere with a distorted shape. The reaction of **L2** with silver nitrate also leads to the formation of a two-dimensional coordination polymer, poly[[μ_3_3,4,8,10,11,13-hexa­hydro-1*H*,6*H*-bis­([1,4]di­thio­cino)[6,7-*b*;6′,7′-*e*]pyrazine-κ^3^ *S*:*S*′:*S*′′](nitrato-κ*O*)silver(I)], [Ag(NO_3_)(C_12_H_16_N_2_S_4_)]_*n*_, (**II**), with the nitrate anion coordinating in a monodentate manner to the silver atom. The silver atom has a fourfold AgOS_3_ coordination sphere with a distorted shape. In the crystals of both complexes, the networks are linked by C—H⋯O hydrogen bonds, forming supra­molecular frameworks. There are additional C—H⋯S contacts present in the supra­molecular framework of **II**.

## Abstract 2

On reaction with silver nitrate the new pyrazine­thio­phanes, 5,7-di­hydro-1*H*,3*H*-dithieno[3,4-*b*:3′,4′-*e*]pyrazine and 3,4,8,10,11,13-hexa­hydro-1*H*,6*H*-bis­([1,4]di­thio­cino)[6,7-*b*:6′,7′-*e*]pyrazine, both form two-dimensional coordination polymers.

## Chemical context   

Ligands with mixed hard and soft binding characters, such as N and S donor atoms, are known to display diverse coordin­ation properties, either by binding selectively to metal centers or by coordination to a wide range of metal cations giving rise to unusual coordination geometries. The title compounds 5,7-di­hydro-1*H*,3*H*-dithieno[3,4-*b*:3′,4′-*e*]pyrazine (**L1**), and 3,4,8,10,11,13-hexa­hydro-1*H*,6*H*-bis­([1,4]di­thio­cino)[6,7-*b*:6′,7′-*e*]pyrazine (**L2**), are new N_2_S_*x*_ (*x* = 2 in **L1** and = 4 in **L2**) ligands designed for the formation of coordination polymers (Assoumatine, 1999 ▸). In **L1**, both the nitro­gen and sulfur potential coordination sites are orientated *exo* to their respective rings. Because of this and the rigidity of the entire mol­ecule, the potential chelating ability appears compromised, as stated by Shimizu and colleagues, who prepared a number of Ag^I^ polymer networks with the benzene analogue of **L1**, 5,7-di­hydro-1*H*,3*H*-benzo[1,2-*c*:4,5-*c′*]di­thio­phene (Shim­izu *et al.*, 1998 ▸; 1999 ▸; Melcer *et al.*, 2001 ▸). A search of the Cambridge Structural Database (Groom *et al.*, 2016 ▸) revealed that **L2** is unique and no benzene analogue or complexes of this analogue have been described. Using the nomenclature of the group of Shim Sung Lee (Siewe *et al.*, 2014 ▸; Kim *et al.*, 2016 ▸, 2018 ▸), **L2** can be described as the bis-*ortho*-l regioisomer. Although, in view of the small size of the macrocycles, it is unlikely that either a *meta*- or a *para*-bis-l regioisomer could be formed.
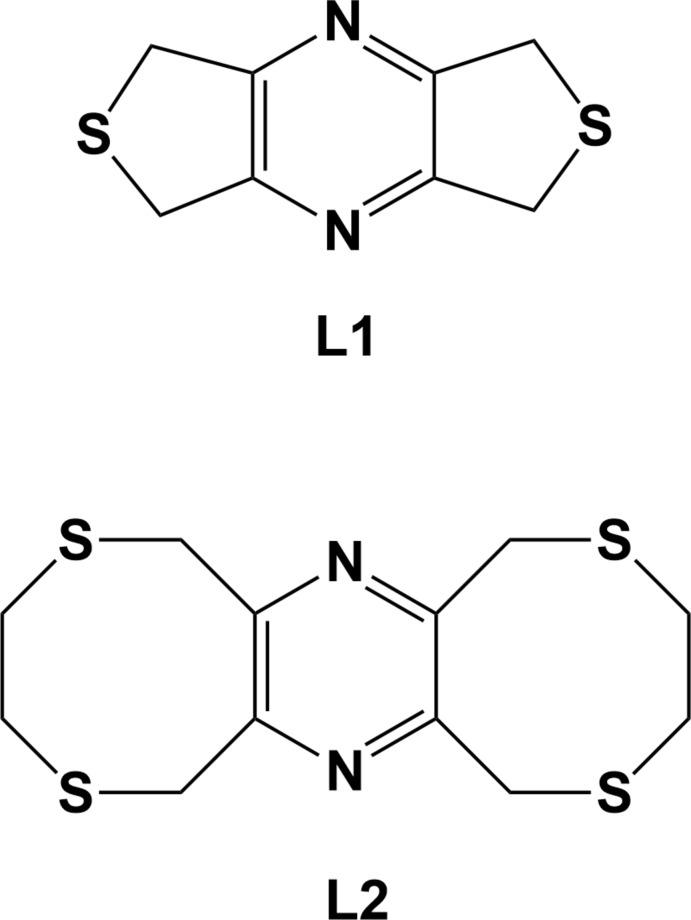


## Structural commentary   

**Figure 1:**
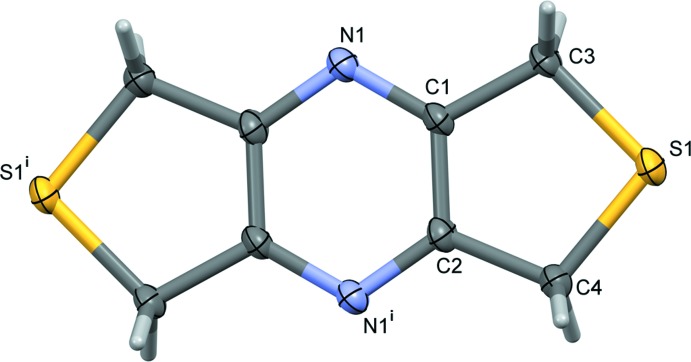
The mol­ecular structure of **L1**, with atom labelling [symmetry code: (i) −*x* + 1, −*y* + 1, −*z* + 1]. Displacement ellipsoids are drawn at the 30% probability level.

The mol­ecular structure of ligand **L1** is illustrated in Fig. 1 ▸. The mol­ecule possesses inversion symmetry and consists of two sulfur atoms linked by a rigid tetra-2,3,5,6-methyl­ene­pyrazine unit. The mol­ecule is planar (r.m.s. deviation = 0.008 Å) with the pyrazine ring being located about a center of symmetry. Both the nitro­gen and sulfur potential coordination sites are orientated *exo* to their respective rings.

**Figure 2:**
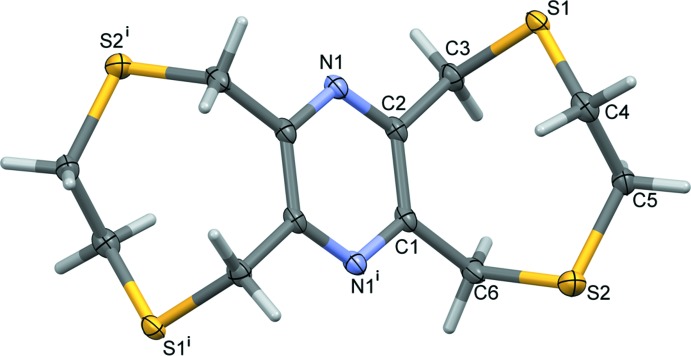
The mol­ecular structure of **L2**, with atom labelling; symmetry code: (i) −*x* + 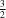, −*y* + 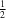, −*z* + 1]. Displacement ellipsoids are drawn at the 30% probability level.

The mol­ecular structure of ligand **L2** is illustrated in Fig. 2 ▸. The mol­ecule also possesses inversion symmetry with the pyrazine ring being located about a center of symmetry. It consists of two S–CH_2_–CH_2_–S chains linked by the central rigid tetra-2,3,5,6-methyl­ene­pyrazine unit, forming eight-membered rings. The configuration of these rings fits best to the definition for a twist-boat-chair (Evans & Boeyens, 1988 ▸; Spek, 2020 ▸), with a pseudo twofold rotation axis bis­ecting the C1—C2 and C4—C5 bonds and their symmetry equivalents. The mol­ecule is step-shaped with six potential sites for coordination.
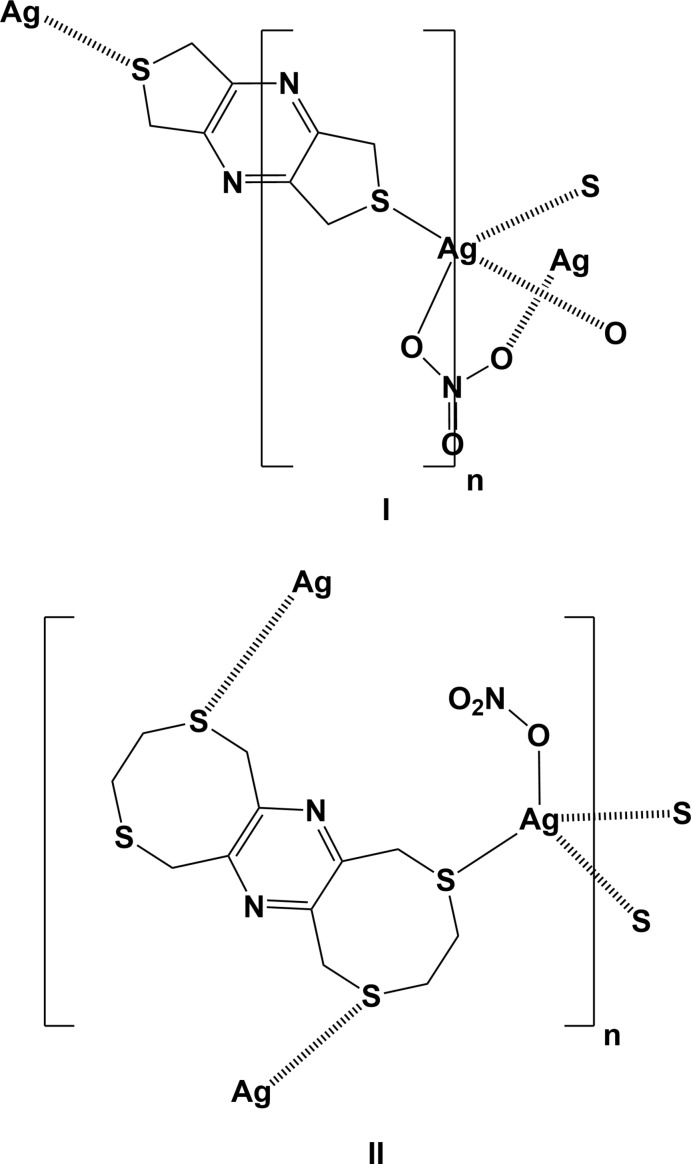


**Figure 3:**
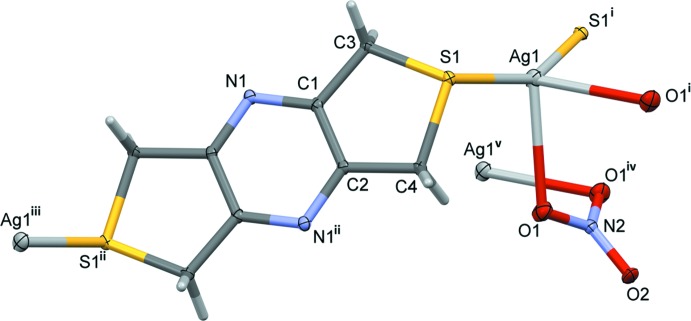
The asymmetric unit of complex **I**, with atom labelling [symmetry codes: (i) −*x* + 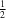, *y*, −*z* + 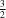; (ii) −*x* + 1, −*y*, −*z* + 2; (iii) −*x* + 1, −*y* + 1, −*z* + 2; (iv) −*x* + 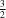, *y*, −*z* + 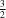; (v) *x* + 1, *y*, *z*]. Displacement ellipsoids are drawn at the 30% probability level.

The reaction of **L1** with silver nitrate leads to the formation of a two-dimensional coordination polymer, (**I)**, with the nitrato anion bridging two equivalent silver atoms (Fig. 3 ▸). Selected bond lengths and bond angles are given in Table 1 ▸. The central pyrazine ring is situated about an inversion center and the silver atom Ag1 and atoms N2 and O2 of the nitrato anion lie on a twofold rotation axis. Atom Ag1 has a fourfold AgO_2_S_2_ coordination sphere with a distorted shape. The fourfold geometry index τ_4_ has a value of 0.74 (τ_4_ = 1 for a perfect tetra­hedral geometry, 0 for a perfect square-planar geometry and 0.85 for perfect trigonal–pyramidal geometry; Yang *et al.*, 2007 ▸). The inter­mediate value of 0.74 tends towards a see-saw arrangement. This seems reasonable in view of the fact that atom Ag1 is located on a twofold rotation axis.

**Figure 4:**
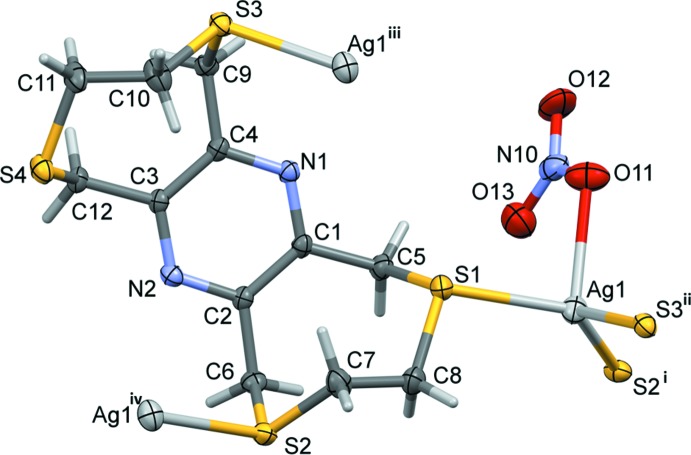
The asymmetric unit of complex **II**, with atom labelling [symmetry codes: (i) −*x* + 1, *y* − 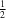, −*z* + 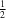; (ii) −*x*, *y* − 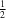, −*z* + 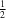); (iii) −*x*, *y* + 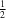, −*z* + 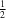; (iv) −*x* + 1, *y* + 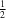, −*z* + 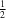]. Displacement ellipsoids are drawn at the 30% probability level.

The reaction of **L2** with silver nitrate also leads to the formation of a two-dimensional coordination polymer (**II**, Fig. 4 ▸). Selected bond lengths and bond angles are given in Table 2 ▸. While the ligand has a step-shape in the solid state with one eight-membered ring directed above the pyrazine ring and the other below (Fig. 2 ▸), in the complex it has a boat shape with both eight-membered rings directed to the same side of the pyrazine ring (Fig. 4 ▸). The configuration of these rings again fits best to the definition for a twist-boat-chair (Evans & Boeyens, 1988 ▸; Spek, 2020 ▸), with a pseudo twofold rotation axis bis­ecting bonds C1—C2 and C7—C8 and bonds C3—C4 and C10—C11. The nitrato anion coordinates to the silver atom in a monodentate manner *via* atom O11 (Fig. 4 ▸, Table 2 ▸). The silver atom Ag1 has a fourfold AgOS_3_ coord­in­ation sphere with a distorted shape. The fourfold geometry index τ_4_ has a value of 0.75, which again tends towards a see-saw arrangement.

The pyrazine N atoms are not involved in coordination to the silver atom in either **I** or **II**; the silver atom prefers coord­ination to the S atoms in both complexes. The role of the nitrato anion in **I** is essential in forming the two-dimensional network, bridging two equivalent silver atoms, while in **II** the nitrato anion coordinates to atom Ag1 in a monodentate manner. There is a significant difference in the Ag—S bond lengths and the Ag—O bond lengths in compounds **I** and **II** (*cf*. Tables 1 ▸ and 2 ▸), which are discussed in §5. *Database survey.*

## Supra­molecular features   

**Figure 5:**
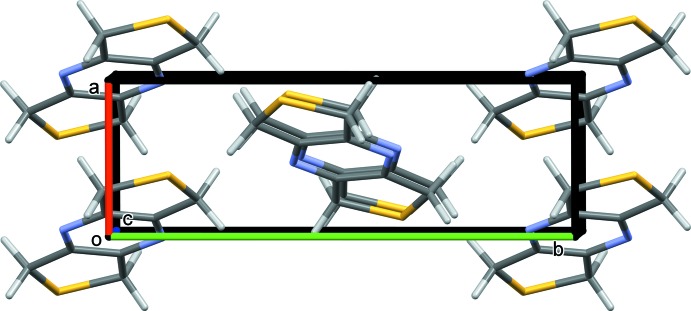
Crystal packing of **L1** viewed along the *c* axis. The mol­ecules stack in columns up the *a* axis.

In the crystals of both **L1** and **L2**, there are no significant inter­molecular inter­actions present (Figs. 5 ▸ and 6 ▸, respectively).

**Figure 7:**
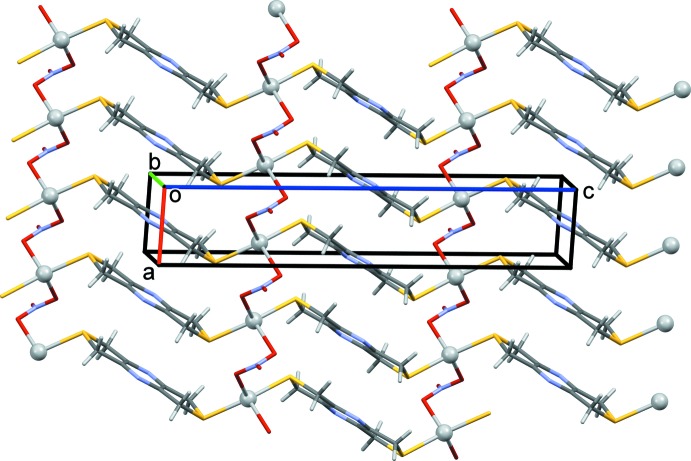
A view along the *b* axis of the crystal packing of complex **I**, illustrating the formation of the metal–organic network. The silver atoms are shown as grey balls.

**Figure 8:**
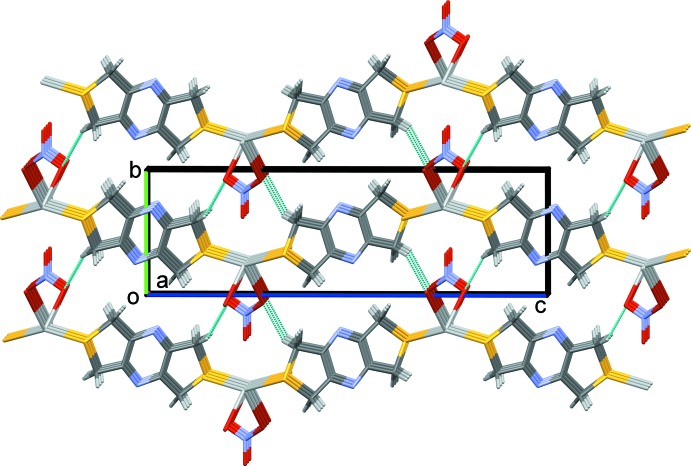
A view along the *a* axis of the crystal packing of complex **I**. The hydrogen bonds are shown as dashed lines (Table 3 ▸).

In the crystal of **I**, the coordination networks lie parallel to the *ac* plane (Fig. 7 ▸) and are linked by C—H⋯O hydrogen bonds, forming a supra­molecular framework (Fig. 8 ▸ and Table 3 ▸).

**Figure 9:**
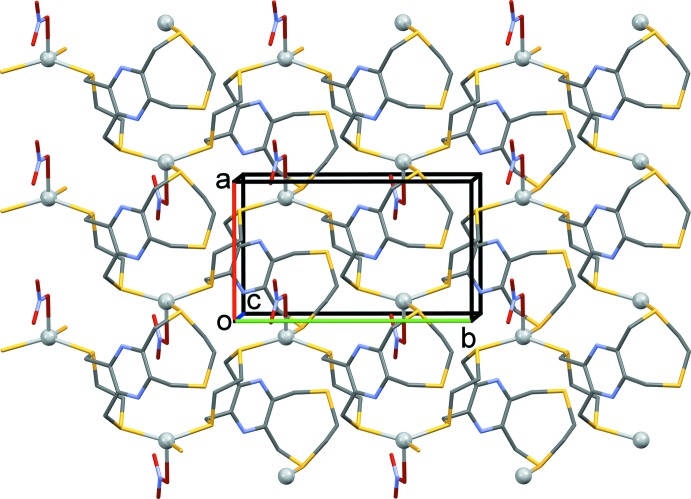
A view along the *c* axis of the crystal packing of complex **II**, illustrating the formation of the metal–organic network. The silver atoms are shown as grey balls. For clarity, the H atoms have been omitted.

**Figure 10:**
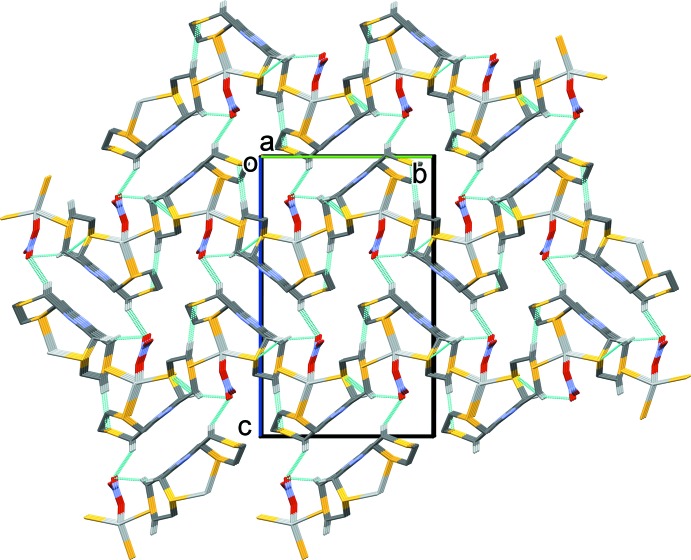
A view along the *a* axis of the crystal packing of complex **II**. The hydrogen bonds are shown as dashed lines (Table 4 ▸). For clarity, only the H atoms involved in these inter­actions have been included.

In the crystal of **II**, the coordination networks lie parallel to the *ab* plane (Fig. 9 ▸). They are linked by C—H⋯O and C—H⋯S hydrogen bonds, forming a supra­molecular framework (Fig. 10 ▸ and Table 4 ▸).

## Hirshfeld surface analysis and two-dimensional fingerprint plots   

The Hirshfeld surface (HS) analyses (Spackman & Jayatilaka, 2009 ▸) and the associated two-dimensional fingerprint plots (McKinnon *et al.*, 2007 ▸) were performed with *CrystalExplorer17* (Turner *et al.*, 2017 ▸) following the protocol of Tiekink and collaborators (Tan *et al.*, 2019 ▸). A summary of the short inter­atomic contacts in **L1** and **L2** is given in Table 5 ▸.

**Figure 11:**
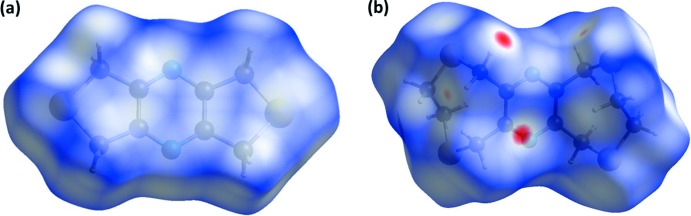
(*a*) The Hirshfeld surface of **L1**, mapped over *d*_norm_ in the colour range 0.0058 to 0.9525 a.u. and (*b*) the Hirshfeld surface of compound **L2**, mapped over *d*_norm_ in the colour range −0.1279 to 1.1192 a.u..

The Hirshfeld surfaces of **L1** and **L2** mapped over *d*_norm_ are given in Fig. 11 ▸ *a* and *b*, respectively. They show that there are no short significant inter­atomic contacts present in the crystal of **L1**, while the red spots indicate that short contacts are significant in the crystal of **L2**.

**Figure 12:**
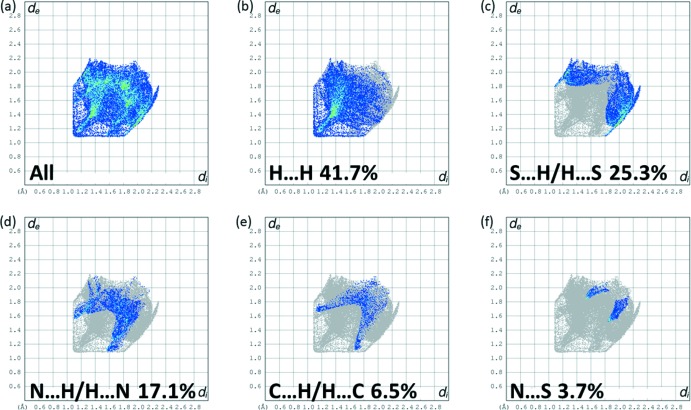
The full two-dimensional fingerprint plot for **L1**, and fingerprint plots delineated into H⋯H, S⋯H/H⋯S, N⋯H/H⋯N, C⋯H/H⋯C, and N⋯S contacts.

**Figure 13:**
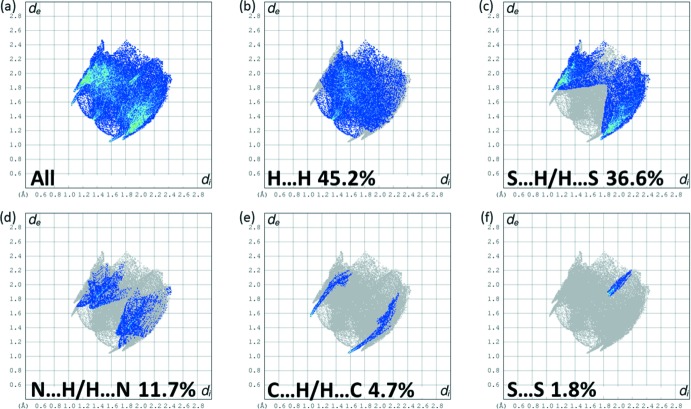
The full two-dimensional fingerprint plot for **L2**, and fingerprint plots delineated into H⋯H, S⋯H/H⋯S, N⋯H/H⋯N, C⋯H/H⋯C, and S⋯S contacts.

The full two-dimensional fingerprint plots for **L1** and **L2** are given in Figs. 12 ▸ and 13 ▸, respectively. The principal inter­molecular inter­actions for **L1** are delineated into the following contacts: H⋯H at 41.7%, S⋯H/H⋯S at 25.3%, N⋯H/H⋯N at 17.1%, C⋯H/H⋯C at 6.5% and N⋯S at 3.7%. For **L2**, the principal inter­molecular inter­actions are delineated into H⋯H contacts at 45.2%, S⋯H/H⋯S at 36.6%, N⋯H/H⋯N at 11.7%, C⋯H/H⋯C at 4.7% and S⋯S at 1.8%. The S⋯H/H⋯S contacts, with the sharp spikes at *d*_e_ + *d*_i_ ≃ 2.9 Å in Fig. 12 ▸ *c* for **L1** and at ≃ 2.80 Å in Fig. 13 ▸ *c* for **L2**, make significant contributions, especially for **L2**, which corresponds to the indications given in Fig. 11 ▸ *b*, the HS of **L2** mapped over *d*_norm_, and in Table 5 ▸. In Fig. 13 ▸ *e* the sharp spikes at *d*_e_ + *d*_i_ ≃ 2.6 Å indicate the significant contribution of the C⋯H/H⋯C contacts in the crystal of **L2**.

## Database survey   

A search of the Cambridge Structural Database (CSD, Version 5.41, last update November 2019; Groom *et al.*, 2016 ▸) for the benzene analogue of **L1**, *i.e*. 5,7-di­hydro-1*H*,3*H*-benzo[1,2-*c*:4,5-*c′*]di­thio­phene, gave ten hits. Five compounds concern silver(I) coordination complexes involving various anions, *viz. catena*-[[μ_2_-5,7-di­hydro-1*H*,3*H*-thieneo(3,4-*f*)(2)benzo­thio­phene]­bis­(aceto­nitrile)­silver(I) hexa­fluorido­phos­phate] (MIZHAE; Melcer *et al.*, 2001 ▸), *catena-*[[μ_3_-1,2:4,5-di­thiolo(*c*)benzene-*S,S,S′*]bis­(aceto­nitrile)­silver(I) tetra­fluor­ido­borate] (NUTBUZ; Shimizu *et al.*, 1998 ▸], *catena-*[[μ_3_-1,2:4,5-di­thiolo(*c*)benzene-*S,S,S′*]benzo­nitrilo­silver tetra­fluor­ido­borate benzo­nitrile solvate] (NUTCAG; Shimizu *et al.*, 1998 ▸)], *catena*-[[μ_3_-benzene-1,2:4,5-bis­(3′,4′-thiol­ane)](*p*-tolylsulfonato)­silver(I) benzene clathrate] (QACYUO; Shimizu *et al.*, 1999 ▸) and *catena*-[bis­(μ_2_-5,7-di­hydro-1*H*,3*H*-thieno(3,4-*f*)(2)benzo­thio­phene)­bis­(*p*-tos­yloxy)disilver(I) benzene solv­ate] (QACYUO01; Melcer *et al.*, 2001 ▸). The latter are two reports of the same compound, *cf*. unit-cell parameters and space group.

The compound MIZHAE is a three-dimensional coordin­ation polymer with a fourfold geometry index τ_4_ value of 0.80 (close to a trigonal–pyramidal geometry) for the silver atom, which has an AgN_2_S_2_coordination sphere. NUTBUZ is a two-dimensional coordination polymer. Here, the silver atom has a fivefold AgN_2_S_3_ coordination sphere with a distorted shape; the fivefold geometry index τ_5_ is 0.77 (τ_5_ = 1 for perfect trigonal–pyramidal geometry and = 0 for perfect square-pyramidal geometry; Addison *et al.*, 1984 ▸). NUTCAG is a two-dimensional coordination polymer with a τ_4_ value of 0.73 for the silver atom, which has an AgNS_3_ coordination sphere. QACYUO (and QACYUO0) is a two-dimensional coordination polymer, with the silver atom having a fourfold AgOS_3_ coordination sphere with a trigonal–pyramidal geometry, the fourfold geometry index τ_4_ being 0.83. The Ag—S bond lengths involving the fourfold coordinated silver atoms vary from 2.4708 (13) Å in NUTCAG to 2.6077 (7) Å in QACYUO/01. The values of the various Ag—S bond lengths in **I** and **II** fall within these limits (see Tables 1 ▸ and 2 ▸). While in the ligand **L1** the five-membered thio­phene rings are planar, in the above mentioned structures and in complex **I** they have envelope configurations with the S atom as the flap.

The nitrate anion can coordinate in at least ten different ways and is extremely useful for designing multi-dimensional coordination polymers, as shown by a search of the CSD. We have previously examined the role of the nitrate anion in the formation of coordination polymers when reporting on the results of the reaction of silver nitrate with some tetra­kis-thio­ether-substituted pyrazine ligands (Assoumatine & Stoeckli-Evans, 2017 ▸). For the two-dimensional coordination polymer (CSD refcode XALPOS) poly[di-μ-nitrato-bis­{μ-2,3,5,6-tetra­kis­[(phenyl­sulfan­yl)meth­yl]pyraz­ine}­disilver(I)] the Ag—O bond lengths vary from 2.507 (4) to 2.551 (4) Å. For the three-dimensional coordination polymer (XALPUY) poly[trinitrato{μ_6_-2,3,5,6-tetra­kis­[(pyridin-2-yl­sulfan­yl)meth­yl]pyrazine}­tris­ilver(I)], the Ag—O bond lengths vary from 2.567 (5) to 2.752 (5) Å. The values observed for **I** and **II**, 2.5849 (15) and 2.492 (3) Å, respectively, are similar to those mentioned above.

A search of the CSD for the benzene analogue of **L2**, or complexes of this analogue, gave zero hits.

## Synthesis and crystallization   

The reagent tetra-2,3,5,6-bromo­methyl-pyrazine (**TBr**) was first synthesized by Ferigo *et al.* (1994 ▸), and its crystal structure has been reported (CSD refcode: TOJXUN; Assoumatine & Stoeckli-Evans, 2014 ▸). The IR spectra for ligands **L1** and **L2**, and for complexes **I** and **II**, are given in Fig. S1 in the supporting information.

**Synthesis of 5,7-di­hydro-1** ***H*** **,3** ***H*** **-dithieno[3,4-** ***b*** **:3′,4′-** ***e*** **]pyrazine (L1)**:

Ligand **L1** was first prepared by the reaction of **TBr** with Na_2_S·9H_2_O, using the procedure of Shimizu *et al.* (1998 ▸). This gave a crude brown solid, which was chromatographed on deactivated silica gel with CH_2_Cl_2_ as eluent to yield 35% of a white solid.

The yield could be increased by as much as 11% using a method similar to that described by Boekelheide *et al.* (1973 ▸). Well-ground Na_2_S·9H_2_O (1.06 g, 4.42 mmol, Aldrich 99%) was dissolved in a solution of MeOH/CH_2_Cl_2_ (100 ml, 1/1 *v*/*v*) in a three-necked flask (500 ml) equipped with a reflux condenser topped by a CaCl_2_ drying tube, an addition funnel (50 ml) and a magnetic stirring bar. To this mixture was added slowly through the addition funnel a solution of **TBr** (1 g, 2.21 mmol) in CH_2_Cl_2_ (25 ml). The reaction mixture was stirred vigorously for 3 h. Removal of the solvent resulted in a brown residue that was extracted into CH_2_Cl_2_ (200 ml), washed with water (3 × 30 ml), dried over anhydrous MgSO4 and then, after filtration, evaporated to dryness. The resultant residue was chromatographed over deactivated silica gel using CH_2_Cl_2_ as eluent. The main eluted fraction was evaporated to give a white solid that was dried under vacuum yielding pure **L1** (m.p. 518–521 K, with decomposition). Colourless rod-like crystals were formed from a concentrated solution of pure **L1** in CH_2_Cl_2_, after standing for one week at 278 K.

^1^H NMR (CDCl_3_, 400 MHz): δ 4.22 (*s*, 8H, Pz–CH_2_–S) ppm. ^13^C NMR (CDCl_3_, 100 MHz): δ 152.30, 34.44 ppm. Analysis for C_8_H_8_N_2_S_2_ (*M*_r_ = 196.30 g mol^−1^). Calculated (%): C 48.95, H 4.11, N 14.27, S 32.67. Found (%): C 49.02, H 4.23, N 14.04, S 32.60. MS (EI, 70 eV), *m*/*z* (%): 196 ([*M* ^+^], 100).

**Synthesis of 3,4,8,10,11,13-hexa­hydro-1** ***H*** **,6** ***H*** **-bis­([1,4]di­thio­cino)[6,7-** ***b*** **:6′,7′-** ***e*** **]pyrazine (L2)**:

A 500 ml three-necked flask was equipped with a reflux condenser, a 50 ml addition funnel, and a magnetic stirring bar. The entire system was purged and kept under a nitro­gen atmosphere using vacuum line techniques. Then well-ground Cs_2_CO_3_ (3.52 g, 10.80 mmol, Fluka 99%) was suspended in DMF (250 ml) in the flask. To this well-stirred suspension was added dropwise through the addition funnel a solution of **TBr** (1 g, 2.21 mmol) and 1,2-ethane­dithiol (0.4 ml, 4.76 mmol, 98%) dissolved in DMF (50 ml), at a rate of about 10 ml h^−1^. The mixture was stirred for a further 20 h and then filtered. The orange filtrate was evaporated under reduced pressure. The residue was extracted into CH_2_Cl_2_ (300 ml) then washed with water (3 × 30 ml), dried over anhydrous MgSO_4_ and then, after filtration, evaporated to dryness. The resultant residue was chromatographed over deactivated silica gel using CH_2_Cl_2_ as eluent. The main eluted fraction was evaporated to give a white solid that was dried under vacuum to obtain 0.35 g (50% yield) of pure **L2** (m.p. 541–544 K, with decomposition). Slow evaporation at room temperature of a solution of **L2** in CHCl_3_ in a 5 mm diameter glass tube gave colourless plate-like crystals.

^1^H NMR (CDCl_3_, 400 MHz): δ 4.08 (*s*, 8H, Pz–CH_2_–S), 2.92 (*s*, 8H, S–CH_2_–CH_2_–S) ppm. ^13^C NMR (CDCl_3_, 100 MHz): δ 151.15, 34.40, 34.09 ppm. Analysis for C_12_H_16_N_2_S_4_ (*M*_r_ = 316.54 g mol^−1^). Calculated (%): C 45.53, H 5.09, N 8.85, S 40.52. Found (%): C 45.34, H 5.30, N 8.68, S 40.33. MS (EI, 70 eV), *m*/*z* (%): 316 ([*M* ^+^], 98.7).

**Synthesis of complex I:**

A solution of **L1** (15 mg, 0.08 mmol) in THF (5 ml) was introduced into a 16 mm diameter glass tube and layered with MeCN (2 ml) as a buffer zone. Then a solution of AgNO_3_ (14 mg, 0.08 mmol) in MeCN (5 ml) was added very gently to avoid possible mixing. The glass tube was sealed and left in the dark at room temperature for at least two weeks, whereupon colourless needle-like crystals of complex **I** were isolated in the buffer zone.

Analysis for C_8_H_8_N_3_O_3_S_2_Ag (*M*_r_ = 366.18 g mol^−1^). Calculated (%): C 26.24, H 2.21, N 11.48, S 17.51. Found (%): C 26.27, H 2.10, N 11.29, S 17.19.

**Synthesis of complex II:**

A solution of **L2** (20 mg, 0.06 mmol) in CH_2_Cl_2_ (10 ml) was introduced into a 16 mm diameter glass tube and layered with MeCN (2 ml) as a buffer zone. Then a solution of AgNO_3_ (10 mg, 0.06 mmol) in MeCN (5 ml) was added very gently to avoid possible mixing. The glass tube was sealed and left in the dark at room temperature for at least three weeks, whereupon thin, colourless plate-like crystals of complex **II** were isolated at the inter­face between the two solutions. No analytical data are available for this complex.

## Refinement   

Crystal data, data collection and structure refinement details are summarized in Table 6 ▸. The C-bound H atoms were included in calculated positions and treated as riding on the parent atoms: C—H = 0.97–0.98 Å with *U*_iso_(H) = 1.2*U*_eq_(C). For **L1**, the rather high *R*_int_ value of 0.159 is due to the poor quality, *viz*. large mosaic spread, of the crystal.

## Supplementary Material

Crystal structure: contains datablock(s) L1, L2, I, II, global. DOI: 10.1107/S205698902000362X/lh5951sup1.cif

Structure factors: contains datablock(s) L1. DOI: 10.1107/S205698902000362X/lh5951L1sup2.hkl

Structure factors: contains datablock(s) L2. DOI: 10.1107/S205698902000362X/lh5951L2sup3.hkl

Structure factors: contains datablock(s) I. DOI: 10.1107/S205698902000362X/lh5951Isup4.hkl

Structure factors: contains datablock(s) II. DOI: 10.1107/S205698902000362X/lh5951IIsup5.hkl

CCDC references: 1989541, 1989540, 1989539, 1989538

Additional supporting information:  crystallographic information; 3D view; checkCIF report

## Figures and Tables

**Figure 6:**
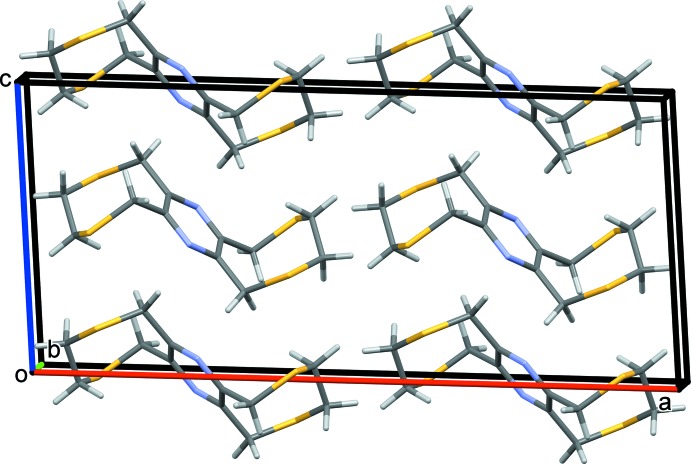
Crystal packing of **L2** viewed along the *b* axis. The mol­ecules stack in columns up the *c* axis.

**Table 1:** Selected geometric parameters (Å, °) for **I** [Chem scheme1]

Ag1—S1	2.4696 (5)	Ag1—O1	2.5849 (15)
			
S1—Ag1—S1^i^	152.57 (2)	S1—Ag1—O1^i^	103.30 (3)
S1—Ag1—O1	97.62 (3)	O1—Ag1—O1^i^	80.24 (7)

Symmetry code: (i) 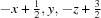.

**Table 2:** Selected geometric parameters (Å, °) for **II** [Chem scheme1]

Ag1—S1	2.5927 (10)	Ag1—S3^ii^	2.5382 (9)
Ag1—S2^i^	2.4760 (10)	Ag1—O11	2.492 (3)
			
S2^i^—Ag1—S1	132.51 (3)	O11—Ag1—S1	93.62 (8)
S3^ii^—Ag1—S1	97.47 (3)	S2^i^—Ag1—O11	97.12 (8)
S2^i^—Ag1—S3^ii^	121.65 (3)	O11—Ag1—S3^ii^	109.25 (8)

Symmetry codes: (i) 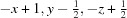; (ii) 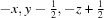.

**Table 3:** Hydrogen-bond geometry (Å, °) for **I** [Chem scheme1]

*D*—H⋯*A*	*D*—H	H⋯*A*	*D*⋯*A*	*D*—H⋯*A*
C3—H3*B*⋯O1^ii^	0.98	2.50	3.379 (2)	150

Symmetry code: (ii) 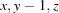.

**Table 4:** Hydrogen-bond geometry (Å, °) for **II** [Chem scheme1]

*D*—H⋯*A*	*D*—H	H⋯*A*	*D*⋯*A*	*D*—H⋯*A*
C5—H5*A*⋯O13	0.97	2.51	3.239 (5)	132
C6—H6*A*⋯O12^iii^	0.97	2.56	3.442 (5)	150
C8—H8*B*⋯S4^i^	0.97	2.74	3.696 (4)	169
C12—H12*B*⋯O12^iv^	0.97	2.37	3.177 (4)	140

Symmetry codes: (i) 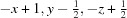; (iii) 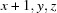; (iv) 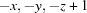.

**Table 5:** Short inter­atomic contacts^*a*^ (Å) in **L1** and **L2**

Atom 1	Atom 2	Length	Length − vdW	Symm. op. 1	Symm. op. 2
**L1**					
H3*A*	H3*A*	2.313	−0.087	1 − *x*, 1 − *y*, −*z*	*x*, *y*, −1 + *z*
H3*B*	C1	2.876	−0.024	*x*, *y*, *z*	−1 + *x*, *y*, *z*
S1	H3*A*	3.000	0.000	1 − *x*, 1 − *y*, −*z*	*x*, *y*, −1 + *z*
H3*B*	N1	2.757	0.007	*x*, *y*, *z*	−1 + *x*, *y*, *z*
S1	C3	3.515	0.015	*x*, *y*, *z*	−*x*, 1 − *y*, 1 − *z*
N1	S1	3.379	0.029	*x*, *y*, *z*	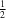 − *x*, − 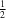 + *y*, 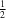 − *z*
S1	C2	3.537	0.037	*x*, *y*, *z*	−1 + *x*, *y*, *z*
H3*B*	H4*B*	2.452	0.052	*x*, *y*, *z*	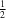 − *x*, − 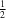 + *y*, 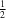 − *z*
C3	H3*A*	2.998	0.098	1 − *x*, 1 − *y*, −*z*	*x*, *y*, −1 + *z*
					
**L2**					
H6*B*	C2	2.699	−0.201	*x*, *y*, *z*	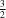 − *x*, − 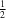 + *y*, 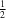 − *z*
S1	H6*A*	2.919	−0.081	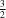 − *x*, 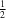 − *y*, 1 − *z*	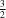 − *x*, − 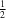 + *y*, 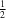 − *z*
S1	H5*A*	2.992	−0.008	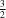 − *x*, 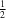 − *y*, 1 − *z*	− 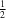 + *x*, − 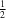 + *y*, *z*
S2	H4*B*	3.017	0.017	*x*, *y*, *z*	*x*, −*y*, − 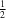 + *z*
S1	C5	3.525	0.025	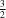 − *x*, 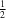 − *y*, 1 − *z*	− 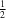 + *x*, − 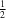 + *y*, *z*

Note: (*a*) Calculated using *Mercury* (Macrae *et al.*, 2020 ▸).

**Table 6:** Experimental details

	**L1**	**L2**	**I**	**II**
Crystal data				
Chemical formula	[Ag(NO_3_)(C_8_H_8_N_2_S_2_)]	[Ag(NO_3_)(C_12_H_16_N_2_S_4_)]	[C_8_H_8_N_2_S_2_]AgNO_3_	[C_12_H_16_N_2_S_4_]AgNO_3_
*M*_r_	196.28	316.51	366.16	486.39
Crystal system, space group	Monoclinic, *P*2_1_/*n*	Monoclinic, *C*2/*c*	Monoclinic, *P*2/*n*	Monoclinic, *P*2_1_/*c*
Temperature (K)	223	223	223	293
*a*, *b*, *c* (Å)	4.1027 (4), 12.1789 (18), 8.1014 (8)	21.1618 (18), 7.0585 (5), 9.5057 (7)	3.8995 (3), 6.3902 (6), 20.5741 (18)	7.0777 (6), 12.0654 (7), 19.5725 (18)
β (°)	95.780 (12)	94.47 (1)	93.121 (9)	90.446 (10)
*V* (Å^3^)	402.74 (8)	1415.55 (19)	511.92 (8)	1671.3 (2)
*Z*	2	4	2	4
Radiation type	Mo *K*α	Mo *K*α	Mo *K*α	Mo *K*α
μ (mm^−1^)	0.60	0.65	2.37	1.72
Crystal size (mm)	0.45 × 0.13 × 0.10	0.40 × 0.30 × 0.10	0.45 × 0.10 × 0.10	0.50 × 0.23 × 0.08

Data collection				
Diffractometer	STOE IPDS 1	STOE IPDS 1	STOE IPDS 1	STOE IPDS 1
Absorption correction	–	–	Multi-scan (*MULABS*; Spek, 2020 ▸)	Multi-scan (*MULABS*; Spek, 2020 ▸)
*T*_min_, *T*_max_	–	–	0.932, 1.000	0.939, 1.000
No. of measured, independent and observed [*I* > 2σ(*I*)] reflections	3025, 744, 590	5086, 1367, 1174	3795, 958, 905	12808, 3222, 2226
*R*_int_	0.159	0.029	0.021	0.051
(sin θ/λ)_max_ (Å^−1^)	0.611	0.615	0.613	0.614

Refinement				
*R*[*F* ^2^ > 2σ(*F* ^2^)], *wR*(*F* ^2^), *S*	0.074, 0.180, 1.03	0.026, 0.071, 1.05	0.016, 0.041, 1.15	0.029, 0.059, 0.85
No. of reflections	744	1367	958	3222
No. of parameters	55	82	79	208
H-atom treatment	H-atom parameters constrained	H-atom parameters constrained	H-atom parameters constrained	H-atom parameters constrained
Δρ_max_, Δρ_min_ (e Å^−3^)	0.77, −0.55	0.31, −0.25	0.31, −0.27	0.61, −0.42

Computer programs: *EXPOSE*, *CELL* and *INTEGRATE* in *IPDS1* (Stoe & Cie, 1998 ▸), *SHELXS97* (Sheldrick, 2008 ▸), *Mercury* (Macrae *et al.*, 2020 ▸), *SHELXL2018/3* (Sheldrick, 2015 ▸), *PLATON* (Spek, 2020 ▸) and *publCIF* (Westrip, 2010 ▸).

## supplementary crystallographic information

### Poly[(µ-5,7-dihydro-1H,3H-dithieno[3,4-b;3',4'-e]pyrazine-κ2S:S')(µ-nitrato-κ2O:O')silver(I)] (L1) Crystal data

**Table:**

[Ag(NO_3_)(C_8_H_8_N_2_S_2_)]	*F*(000) = 204
*M**_r_* = 196.28	*D*_x_ = 1.619 Mg m^−^^3^
Monoclinic, *P*2_1_/*n*	Mo *K*α radiation, λ = 0.71073 Å
*a* = 4.1027 (4) Å	Cell parameters from 3541 reflections
*b* = 12.1789 (18) Å	θ = 3.1–25.7°
*c* = 8.1014 (8) Å	µ = 0.60 mm^−^^1^
β = 95.780 (12)°	*T* = 223 K
*V* = 402.74 (8) Å^3^	Rod, colourless
*Z* = 2	0.45 × 0.13 × 0.10 mm

### Poly[(µ-5,7-dihydro-1H,3H-dithieno[3,4-b;3',4'-e]pyrazine-κ2S:S')(µ-nitrato-κ2O:O')silver(I)] (L1) Data collection

**Table:**

STOE IPDS 1 diffractometer	590 reflections with *I* > 2σ(*I*)
Radiation source: fine-focus sealed tube	*R*_int_ = 0.159
Plane graphite monochromator	θ_max_ = 25.8°, θ_min_ = 3.0°
φ rotation scans	*h* = −4→4
3025 measured reflections	*k* = −14→14
744 independent reflections	*l* = −9→9

### Poly[(µ-5,7-dihydro-1H,3H-dithieno[3,4-b;3',4'-e]pyrazine-κ2S:S')(µ-nitrato-κ2O:O')silver(I)] (L1) Refinement

**Table:**

Refinement on *F*^2^	Primary atom site location: structure-invariant direct methods
Least-squares matrix: full	Secondary atom site location: difference Fourier map
*R*[*F*^2^ > 2σ(*F*^2^)] = 0.074	Hydrogen site location: inferred from neighbouring sites
*wR*(*F*^2^) = 0.180	H-atom parameters constrained
*S* = 1.03	*w* = 1/[σ^2^(*F*_o_^2^) + (0.1296*P*)^2^] where *P* = (*F*_o_^2^ + 2*F*_c_^2^)/3
744 reflections	(Δ/σ)_max_ < 0.001
55 parameters	Δρ_max_ = 0.77 e Å^−^^3^
0 restraints	Δρ_min_ = −0.55 e Å^−^^3^

### Poly[(µ-5,7-dihydro-1H,3H-dithieno[3,4-b;3',4'-e]pyrazine-κ2S:S')(µ-nitrato-κ2O:O')silver(I)] (L1) Special details

**Table:**

Geometry. All esds (except the esd in the dihedral angle between two l.s. planes) are estimated using the full covariance matrix. The cell esds are taken into account individually in the estimation of esds in distances, angles and torsion angles; correlations between esds in cell parameters are only used when they are defined by crystal symmetry. An approximate (isotropic) treatment of cell esds is used for estimating esds involving l.s. planes.

### Poly[(µ-5,7-dihydro-1H,3H-dithieno[3,4-b;3',4'-e]pyrazine-κ2S:S')(µ-nitrato-κ2O:O')silver(I)] (L1) Fractional atomic coordinates and isotropic or equivalent isotropic displacement parameters (Å^2^)

**Table:**

	*x*	*y*	*z*	*U*_iso_*/*U*_eq_	
S1	0.1126 (2)	0.62437 (7)	0.34804 (9)	0.0363 (5)	
N1	0.4717 (8)	0.3945 (2)	0.0713 (4)	0.0320 (7)	
C1	0.3670 (8)	0.4860 (3)	0.1411 (3)	0.0296 (9)	
C2	0.3971 (9)	0.5902 (3)	0.0708 (4)	0.0303 (8)	
C3	0.2147 (9)	0.4824 (3)	0.3009 (3)	0.0341 (9)	
H3A	0.368695	0.451919	0.389472	0.041*	
H3B	0.016898	0.436835	0.289632	0.041*	
C4	0.2721 (10)	0.6844 (3)	0.1673 (4)	0.0356 (9)	
H4A	0.098963	0.724060	0.099563	0.043*	
H4B	0.450057	0.735867	0.201262	0.043*	

### Poly[(µ-5,7-dihydro-1H,3H-dithieno[3,4-b;3',4'-e]pyrazine-κ2S:S')(µ-nitrato-κ2O:O')silver(I)] (L1) Atomic displacement parameters (Å^2^)

**Table:**

	*U*^11^	*U*^22^	*U*^33^	*U*^12^	*U*^13^	*U*^23^
S1	0.0477 (8)	0.0408 (6)	0.0219 (6)	0.0041 (3)	0.0106 (4)	−0.0011 (3)
N1	0.0412 (18)	0.0333 (14)	0.0219 (15)	0.0007 (11)	0.0049 (11)	0.0019 (10)
C1	0.034 (2)	0.0354 (18)	0.0197 (17)	−0.0011 (12)	0.0062 (13)	0.0012 (11)
C2	0.039 (2)	0.0349 (17)	0.0161 (15)	0.0014 (14)	0.0007 (12)	−0.0031 (12)
C3	0.048 (2)	0.0363 (17)	0.0187 (18)	0.0023 (14)	0.0061 (15)	0.0031 (12)
C4	0.048 (2)	0.0328 (17)	0.0268 (16)	0.0012 (14)	0.0096 (15)	0.0010 (12)

### Poly[(µ-5,7-dihydro-1H,3H-dithieno[3,4-b;3',4'-e]pyrazine-κ2S:S')(µ-nitrato-κ2O:O')silver(I)] (L1) Geometric parameters (Å, º)

**Table:**

S1—C4	1.817 (4)	C2—C4	1.507 (5)
S1—C3	1.828 (3)	C3—H3A	0.9800
N1—C2^i^	1.332 (4)	C3—H3B	0.9800
N1—C1	1.340 (4)	C4—H4A	0.9800
C1—C2	1.401 (5)	C4—H4B	0.9800
C1—C3	1.494 (4)		
			
C4—S1—C3	95.95 (14)	S1—C3—H3A	110.5
C2^i^—N1—C1	115.0 (3)	C1—C3—H3B	110.5
N1—C1—C2	122.5 (3)	S1—C3—H3B	110.5
N1—C1—C3	121.4 (3)	H3A—C3—H3B	108.7
C2—C1—C3	116.1 (3)	C2—C4—S1	106.3 (2)
N1^i^—C2—C1	122.5 (3)	C2—C4—H4A	110.5
N1^i^—C2—C4	122.0 (3)	S1—C4—H4A	110.5
C1—C2—C4	115.5 (3)	C2—C4—H4B	110.5
C1—C3—S1	106.1 (2)	S1—C4—H4B	110.5
C1—C3—H3A	110.5	H4A—C4—H4B	108.7
			
C2^i^—N1—C1—C2	−0.8 (5)	N1—C1—C3—S1	179.8 (3)
C2^i^—N1—C1—C3	−179.9 (3)	C2—C1—C3—S1	0.7 (4)
N1—C1—C2—N1^i^	0.8 (6)	C4—S1—C3—C1	−1.1 (3)
C3—C1—C2—N1^i^	180.0 (3)	N1^i^—C2—C4—S1	179.2 (3)
N1—C1—C2—C4	−178.9 (3)	C1—C2—C4—S1	−1.0 (4)
C3—C1—C2—C4	0.3 (5)	C3—S1—C4—C2	1.2 (3)

Symmetry code: (i) −*x*+1, −*y*+1, −*z*.

### Poly[[µ33,4,8,10,11,13-hexahydro-1H,6H-bis([1,4]dithiocino)[6,7-b;6',7'-e]pyrazine-κ3S:S':S''](nitrato-κO)silver(I)] (L2) Crystal data

**Table:**

[Ag(NO_3_)(C_12_H_16_N_2_S_4_)]	*F*(000) = 664
*M**_r_* = 316.51	*D*_x_ = 1.485 Mg m^−^^3^
Monoclinic, *C*2/*c*	Mo *K*α radiation, λ = 0.71073 Å
*a* = 21.1618 (18) Å	Cell parameters from 5000 reflections
*b* = 7.0585 (5) Å	θ = 3.0–25.9°
*c* = 9.5057 (7) Å	µ = 0.65 mm^−^^1^
β = 94.47 (1)°	*T* = 223 K
*V* = 1415.55 (19) Å^3^	Colourless, plate
*Z* = 4	0.40 × 0.30 × 0.10 mm

### Poly[[µ33,4,8,10,11,13-hexahydro-1H,6H-bis([1,4]dithiocino)[6,7-b;6',7'-e]pyrazine-κ3S:S':S''](nitrato-κO)silver(I)] (L2) Data collection

**Table:**

STOE IPDS 1 diffractometer	1174 reflections with *I* > 2σ(*I*)
Radiation source: fine-focus sealed tube	*R*_int_ = 0.029
Plane graphite monochromator	θ_max_ = 25.9°, θ_min_ = 3.0°
φ rotation scans	*h* = −25→25
5086 measured reflections	*k* = −8→8
1367 independent reflections	*l* = −11→11

### Poly[[µ33,4,8,10,11,13-hexahydro-1H,6H-bis([1,4]dithiocino)[6,7-b;6',7'-e]pyrazine-κ3S:S':S''](nitrato-κO)silver(I)] (L2) Refinement

**Table:**

Refinement on *F*^2^	Primary atom site location: structure-invariant direct methods
Least-squares matrix: full	Secondary atom site location: difference Fourier map
*R*[*F*^2^ > 2σ(*F*^2^)] = 0.026	Hydrogen site location: inferred from neighbouring sites
*wR*(*F*^2^) = 0.071	H-atom parameters constrained
*S* = 1.05	*w* = 1/[σ^2^(*F*_o_^2^) + (0.0427*P*)^2^ + 0.461*P*] where *P* = (*F*_o_^2^ + 2*F*_c_^2^)/3
1367 reflections	(Δ/σ)_max_ = 0.001
82 parameters	Δρ_max_ = 0.31 e Å^−^^3^
0 restraints	Δρ_min_ = −0.25 e Å^−^^3^

### Poly[[µ33,4,8,10,11,13-hexahydro-1H,6H-bis([1,4]dithiocino)[6,7-b;6',7'-e]pyrazine-κ3S:S':S''](nitrato-κO)silver(I)] (L2) Special details

**Table:**

Geometry. All esds (except the esd in the dihedral angle between two l.s. planes) are estimated using the full covariance matrix. The cell esds are taken into account individually in the estimation of esds in distances, angles and torsion angles; correlations between esds in cell parameters are only used when they are defined by crystal symmetry. An approximate (isotropic) treatment of cell esds is used for estimating esds involving l.s. planes.

### Poly[[µ33,4,8,10,11,13-hexahydro-1H,6H-bis([1,4]dithiocino)[6,7-b;6',7'-e]pyrazine-κ3S:S':S''](nitrato-κO)silver(I)] (L2) Fractional atomic coordinates and isotropic or equivalent isotropic displacement parameters (Å^2^)

**Table:**

	*x*	*y*	*z*	*U*_iso_*/*U*_eq_	
S1	0.90795 (2)	0.54315 (6)	0.52645 (5)	0.03312 (15)	
S2	0.89795 (2)	0.01187 (6)	0.34452 (5)	0.03987 (16)	
N1	0.75497 (6)	0.42931 (19)	0.55990 (14)	0.0268 (3)	
C1	0.78592 (7)	0.1989 (2)	0.39521 (15)	0.0253 (3)	
C2	0.79056 (6)	0.3798 (2)	0.45558 (15)	0.0246 (3)	
C3	0.83585 (8)	0.5281 (2)	0.41003 (18)	0.0303 (4)	
H3A	0.814637	0.651638	0.407231	0.036*	
H3B	0.847038	0.498872	0.314291	0.036*	
C4	0.93325 (8)	0.2996 (2)	0.54130 (17)	0.0323 (4)	
H4A	0.970847	0.293157	0.608187	0.039*	
H4B	0.899694	0.225955	0.581154	0.039*	
C5	0.94907 (7)	0.2061 (2)	0.40278 (18)	0.0319 (4)	
H5A	0.992794	0.159497	0.414165	0.038*	
H5B	0.947061	0.302840	0.328648	0.038*	
C6	0.82558 (7)	0.1326 (3)	0.27995 (17)	0.0332 (4)	
H6A	0.836729	0.242364	0.223784	0.040*	
H6B	0.800162	0.046620	0.217500	0.040*	

### Poly[[µ33,4,8,10,11,13-hexahydro-1H,6H-bis([1,4]dithiocino)[6,7-b;6',7'-e]pyrazine-κ3S:S':S''](nitrato-κO)silver(I)] (L2) Atomic displacement parameters (Å^2^)

**Table:**

	*U*^11^	*U*^22^	*U*^33^	*U*^12^	*U*^13^	*U*^23^
S1	0.0246 (2)	0.0341 (2)	0.0413 (3)	−0.01170 (15)	0.00595 (17)	−0.01108 (17)
S2	0.0336 (3)	0.0307 (2)	0.0562 (3)	−0.00329 (16)	0.0090 (2)	−0.01143 (19)
N1	0.0215 (6)	0.0287 (7)	0.0300 (7)	−0.0059 (5)	0.0004 (5)	−0.0012 (5)
C1	0.0196 (7)	0.0326 (8)	0.0231 (8)	−0.0055 (6)	−0.0017 (6)	0.0008 (6)
C2	0.0186 (7)	0.0295 (8)	0.0252 (8)	−0.0058 (6)	−0.0013 (6)	0.0032 (6)
C3	0.0266 (8)	0.0289 (8)	0.0356 (9)	−0.0071 (6)	0.0044 (6)	0.0035 (7)
C4	0.0292 (8)	0.0375 (9)	0.0296 (8)	−0.0063 (7)	−0.0008 (6)	0.0047 (7)
C5	0.0226 (7)	0.0330 (8)	0.0402 (9)	−0.0016 (6)	0.0039 (6)	−0.0010 (7)
C6	0.0281 (8)	0.0435 (9)	0.0283 (8)	−0.0109 (7)	0.0038 (6)	−0.0085 (7)

### Poly[[µ33,4,8,10,11,13-hexahydro-1H,6H-bis([1,4]dithiocino)[6,7-b;6',7'-e]pyrazine-κ3S:S':S''](nitrato-κO)silver(I)] (L2) Geometric parameters (Å, º)

**Table:**

S1—C4	1.8025 (17)	C3—H3A	0.9800
S1—C3	1.8168 (17)	C3—H3B	0.9800
S2—C5	1.8062 (16)	C4—C5	1.533 (2)
S2—C6	1.8171 (18)	C4—H4A	0.9800
N1—C2	1.338 (2)	C4—H4B	0.9800
N1—C1^i^	1.3443 (19)	C5—H5A	0.9800
C1—C2	1.401 (2)	C5—H5B	0.9800
C1—C6	1.506 (2)	C6—H6A	0.9800
C2—C3	1.506 (2)	C6—H6B	0.9800
			
C4—S1—C3	102.85 (8)	S1—C4—H4A	108.5
C5—S2—C6	102.50 (8)	C5—C4—H4B	108.5
C2—N1—C1^i^	118.24 (14)	S1—C4—H4B	108.5
N1^i^—C1—C2	120.67 (14)	H4A—C4—H4B	107.5
N1^i^—C1—C6	115.54 (14)	C4—C5—S2	115.13 (11)
C2—C1—C6	123.79 (13)	C4—C5—H5A	108.5
N1—C2—C1	121.09 (13)	S2—C5—H5A	108.5
N1—C2—C3	116.07 (14)	C4—C5—H5B	108.5
C1—C2—C3	122.83 (14)	S2—C5—H5B	108.5
C2—C3—S1	112.86 (11)	H5A—C5—H5B	107.5
C2—C3—H3A	109.0	C1—C6—S2	113.75 (11)
S1—C3—H3A	109.0	C1—C6—H6A	108.8
C2—C3—H3B	109.0	S2—C6—H6A	108.8
S1—C3—H3B	109.0	C1—C6—H6B	108.8
H3A—C3—H3B	107.8	S2—C6—H6B	108.8
C5—C4—S1	115.28 (11)	H6A—C6—H6B	107.7
C5—C4—H4A	108.5		
			
C1^i^—N1—C2—C1	−0.5 (2)	C4—S1—C3—C2	49.78 (13)
C1^i^—N1—C2—C3	−179.60 (14)	C3—S1—C4—C5	61.99 (13)
N1^i^—C1—C2—N1	0.6 (2)	S1—C4—C5—S2	−115.40 (11)
C6—C1—C2—N1	−178.56 (14)	C6—S2—C5—C4	74.34 (13)
N1^i^—C1—C2—C3	179.55 (14)	N1^i^—C1—C6—S2	−86.52 (14)
C6—C1—C2—C3	0.4 (2)	C2—C1—C6—S2	92.63 (17)
N1—C2—C3—S1	80.06 (15)	C5—S2—C6—C1	−77.62 (13)
C1—C2—C3—S1	−98.99 (15)		

Symmetry code: (i) −*x*+3/2, −*y*+1/2, −*z*+1.

### Poly[[µ33,4,8,10,11,13-hexahydro-1H,6H-bis([1,4]dithiocino)[6,7-b;6',7'-e]pyrazine-κ3S:S':S''](nitrato-κO)silver(I)] (L2) Hydrogen-bond geometry (Å, º)

**Table:**

*D*—H···*A*	*D*—H	H···*A*	*D*···*A*	*D*—H···*A*
C4—H4*B*···S2^ii^	0.98	3.02	3.7465 (17)	132
C5—H5*A*···S1^iii^	0.98	2.99	3.5248 (16)	115
C6—H6*A*···S1^iv^	0.98	2.92	3.8389 (18)	157

Symmetry codes: (ii) *x*, −*y*, *z*+1/2; (iii) −*x*+2, −*y*+1, −*z*+1; (iv) *x*, −*y*+1, *z*−1/2.

### (I) Crystal data

**Table:**

[C_8_H_8_N_2_S_2_]AgNO_3_	*F*(000) = 360
*M**_r_* = 366.16	*D*_x_ = 2.375 Mg m^−^^3^
Monoclinic, *P*2/*n*	Mo *K*α radiation, λ = 0.71073 Å
*a* = 3.8995 (3) Å	Cell parameters from 5000 reflections
*b* = 6.3902 (6) Å	θ = 3.2–25.8°
*c* = 20.5741 (18) Å	µ = 2.37 mm^−^^1^
β = 93.121 (9)°	*T* = 223 K
*V* = 511.92 (8) Å^3^	Needle, colourless
*Z* = 2	0.45 × 0.10 × 0.10 mm

### (I) Data collection

**Table:**

STOE IPDS 1 diffractometer	958 independent reflections
Radiation source: fine-focus sealed tube	905 reflections with *I* > 2σ(*I*)
Plane graphite monochromator	*R*_int_ = 0.021
φ rotation scans	θ_max_ = 25.8°, θ_min_ = 3.2°
Absorption correction: multi-scan (MULABS; Spek, 2020)	*h* = −4→4
*T*_min_ = 0.932, *T*_max_ = 1.000	*k* = −7→7
3795 measured reflections	*l* = −25→25

### (I) Refinement

**Table:**

Refinement on *F*^2^	Primary atom site location: structure-invariant direct methods
Least-squares matrix: full	Secondary atom site location: difference Fourier map
*R*[*F*^2^ > 2σ(*F*^2^)] = 0.016	Hydrogen site location: inferred from neighbouring sites
*wR*(*F*^2^) = 0.041	H-atom parameters constrained
*S* = 1.15	*w* = 1/[σ^2^(*F*_o_^2^) + (0.020*P*)^2^ + 0.3928*P*] where *P* = (*F*_o_^2^ + 2*F*_c_^2^)/3
958 reflections	(Δ/σ)_max_ = 0.001
79 parameters	Δρ_max_ = 0.31 e Å^−^^3^
0 restraints	Δρ_min_ = −0.27 e Å^−^^3^

### (I) Special details

**Table:**

Geometry. All esds (except the esd in the dihedral angle between two l.s. planes) are estimated using the full covariance matrix. The cell esds are taken into account individually in the estimation of esds in distances, angles and torsion angles; correlations between esds in cell parameters are only used when they are defined by crystal symmetry. An approximate (isotropic) treatment of cell esds is used for estimating esds involving l.s. planes.

### (I) Fractional atomic coordinates and isotropic or equivalent isotropic displacement parameters (Å^2^)

**Table:**

	*x*	*y*	*z*	*U*_iso_*/*U*_eq_	
Ag1	0.250000	0.73580 (3)	0.750000	0.02211 (9)	
S1	−0.02263 (12)	0.64417 (7)	0.85176 (2)	0.01302 (12)	
O1	0.6178 (4)	1.0451 (2)	0.79516 (7)	0.0274 (3)	
O2	0.750000	1.3371 (3)	0.750000	0.0262 (5)	
N1	0.4947 (4)	0.2874 (2)	0.97975 (7)	0.0136 (3)	
N2	0.750000	1.1423 (3)	0.750000	0.0157 (5)	
C1	0.3461 (5)	0.4408 (3)	0.94406 (8)	0.0126 (4)	
C2	0.3516 (5)	0.6499 (3)	0.96379 (8)	0.0124 (4)	
C3	0.1666 (5)	0.3949 (3)	0.87926 (9)	0.0143 (4)	
H3A	−0.012208	0.288847	0.883653	0.017*	
H3B	0.330073	0.344062	0.848344	0.017*	
C4	0.1801 (5)	0.8040 (3)	0.91737 (9)	0.0148 (4)	
H4A	0.349176	0.899858	0.900192	0.018*	
H4B	0.007436	0.886025	0.939085	0.018*	

### (I) Atomic displacement parameters (Å^2^)

**Table:**

	*U*^11^	*U*^22^	*U*^33^	*U*^12^	*U*^13^	*U*^23^
Ag1	0.03440 (16)	0.01965 (13)	0.01297 (13)	0.000	0.00761 (9)	0.000
S1	0.0137 (2)	0.0144 (2)	0.0109 (2)	0.00041 (16)	−0.00013 (16)	0.00061 (15)
O1	0.0383 (9)	0.0218 (7)	0.0230 (7)	−0.0058 (7)	0.0090 (7)	0.0021 (6)
O2	0.0451 (14)	0.0098 (9)	0.0228 (10)	0.000	−0.0058 (9)	0.000
N1	0.0155 (8)	0.0134 (7)	0.0118 (7)	−0.0009 (6)	0.0015 (6)	0.0001 (6)
N2	0.0175 (12)	0.0146 (11)	0.0144 (11)	0.000	−0.0040 (9)	0.000
C1	0.0127 (9)	0.0145 (9)	0.0106 (8)	−0.0012 (7)	0.0021 (6)	0.0009 (7)
C2	0.0136 (9)	0.0134 (9)	0.0104 (8)	−0.0005 (7)	0.0020 (7)	0.0005 (7)
C3	0.0175 (9)	0.0125 (8)	0.0126 (8)	0.0007 (7)	−0.0014 (7)	−0.0010 (7)
C4	0.0198 (10)	0.0128 (8)	0.0114 (8)	0.0006 (7)	−0.0015 (7)	−0.0013 (7)

### (I) Geometric parameters (Å, º)

**Table:**

Ag1—S1	2.4696 (5)	N1—C2^ii^	1.339 (2)
Ag1—S1^i^	2.4696 (5)	C1—C2	1.397 (3)
Ag1—O1	2.5849 (15)	C1—C3	1.501 (2)
Ag1—O1^i^	2.5849 (15)	C2—C4	1.503 (3)
S1—C3	1.8322 (19)	C3—H3A	0.9800
S1—C4	1.8368 (19)	C3—H3B	0.9800
O1—N2	1.2517 (18)	C4—H4A	0.9800
O2—N2	1.245 (3)	C4—H4B	0.9800
N1—C1	1.337 (2)		
			
S1—Ag1—S1^i^	152.57 (2)	C2—C1—C3	116.38 (16)
S1—Ag1—O1	97.62 (3)	N1^ii^—C2—C1	122.49 (17)
S1^i^—Ag1—O1	103.30 (4)	N1^ii^—C2—C4	121.21 (16)
S1—Ag1—O1^i^	103.30 (3)	C1—C2—C4	116.29 (16)
S1^i^—Ag1—O1^i^	97.62 (3)	C1—C3—S1	105.36 (12)
O1—Ag1—O1^i^	80.24 (7)	C1—C3—H3A	110.7
C3—S1—C4	96.12 (9)	S1—C3—H3A	110.7
C3—S1—Ag1	106.45 (6)	C1—C3—H3B	110.7
C4—S1—Ag1	107.66 (6)	S1—C3—H3B	110.7
N2—O1—Ag1	110.82 (11)	H3A—C3—H3B	108.8
C1—N1—C2^ii^	114.66 (16)	C2—C4—S1	105.17 (12)
O2—N2—O1	119.74 (11)	C2—C4—H4A	110.7
O2—N2—O1^iii^	119.74 (11)	S1—C4—H4A	110.7
O1—N2—O1^iii^	120.5 (2)	C2—C4—H4B	110.7
N1—C1—C2	122.85 (17)	S1—C4—H4B	110.7
N1—C1—C3	120.77 (16)	H4A—C4—H4B	108.8
			
Ag1—O1—N2—O2	136.46 (5)	N1—C1—C3—S1	175.73 (14)
Ag1—O1—N2—O1^iii^	−43.54 (5)	C2—C1—C3—S1	−5.08 (19)
C2^ii^—N1—C1—C2	0.3 (3)	C4—S1—C3—C1	7.26 (14)
C2^ii^—N1—C1—C3	179.41 (16)	Ag1—S1—C3—C1	117.73 (11)
N1—C1—C2—N1^ii^	−0.3 (3)	N1^ii^—C2—C4—S1	−175.17 (14)
C3—C1—C2—N1^ii^	−179.47 (16)	C1—C2—C4—S1	5.9 (2)
N1—C1—C2—C4	178.56 (17)	C3—S1—C4—C2	−7.54 (14)
C3—C1—C2—C4	−0.6 (2)	Ag1—S1—C4—C2	−116.99 (11)

Symmetry codes: (i) −*x*+1/2, *y*, −*z*+3/2; (ii) −*x*+1, −*y*+1, −*z*+2; (iii) −*x*+3/2, *y*, −*z*+3/2.

### (I) Hydrogen-bond geometry (Å, º)

**Table:**

*D*—H···*A*	*D*—H	H···*A*	*D*···*A*	*D*—H···*A*
C3—H3*B*···O1^iv^	0.98	2.50	3.379 (2)	150
C4—H4*A*···O1	0.98	2.62	3.475 (2)	146

Symmetry code: (iv) *x*, *y*−1, *z*.

### (II) Crystal data

**Table:**

[C_12_H_16_N_2_S_4_]AgNO_3_	*F*(000) = 976
*M**_r_* = 486.39	*D*_x_ = 1.933 Mg m^−^^3^
Monoclinic, *P*2_1_/*c*	Mo *K*α radiation, λ = 0.71073 Å
*a* = 7.0777 (6) Å	Cell parameters from 5000 reflections
*b* = 12.0654 (7) Å	θ = 2.1–25.9°
*c* = 19.5725 (18) Å	µ = 1.72 mm^−^^1^
β = 90.446 (10)°	*T* = 293 K
*V* = 1671.3 (2) Å^3^	Plate, colourless
*Z* = 4	0.50 × 0.23 × 0.08 mm

### (II) Data collection

**Table:**

STOE IPDS 1 diffractometer	3222 independent reflections
Radiation source: fine-focus sealed tube	2226 reflections with *I* > 2σ(*I*)
Plane graphite monochromator	*R*_int_ = 0.051
φ rotation scans	θ_max_ = 25.9°, θ_min_ = 2.7°
Absorption correction: multi-scan (MULABS; Spek, 2020)	*h* = −8→8
*T*_min_ = 0.939, *T*_max_ = 1.000	*k* = −14→14
12808 measured reflections	*l* = −23→23

### (II) Refinement

**Table:**

Refinement on *F*^2^	Primary atom site location: structure-invariant direct methods
Least-squares matrix: full	Secondary atom site location: difference Fourier map
*R*[*F*^2^ > 2σ(*F*^2^)] = 0.029	Hydrogen site location: inferred from neighbouring sites
*wR*(*F*^2^) = 0.059	H-atom parameters constrained
*S* = 0.85	*w* = 1/[σ^2^(*F*_o_^2^) + (0.0288*P*)^2^] where *P* = (*F*_o_^2^ + 2*F*_c_^2^)/3
3222 reflections	(Δ/σ)_max_ < 0.001
208 parameters	Δρ_max_ = 0.61 e Å^−^^3^
0 restraints	Δρ_min_ = −0.42 e Å^−^^3^

### (II) Special details

**Table:**

Geometry. All esds (except the esd in the dihedral angle between two l.s. planes) are estimated using the full covariance matrix. The cell esds are taken into account individually in the estimation of esds in distances, angles and torsion angles; correlations between esds in cell parameters are only used when they are defined by crystal symmetry. An approximate (isotropic) treatment of cell esds is used for estimating esds involving l.s. planes.

### (II) Fractional atomic coordinates and isotropic or equivalent isotropic displacement parameters (Å^2^)

**Table:**

	*x*	*y*	*z*	*U*_iso_*/*U*_eq_	
Ag1	0.13078 (4)	−0.29440 (2)	0.19436 (2)	0.04241 (10)	
S1	0.24146 (12)	−0.10620 (8)	0.24454 (5)	0.0373 (2)	
S2	0.77716 (12)	0.01162 (8)	0.28097 (4)	0.0348 (2)	
S3	−0.06576 (11)	0.27177 (7)	0.42666 (4)	0.0339 (2)	
S4	0.47060 (13)	0.37232 (8)	0.47535 (5)	0.0393 (2)	
N1	0.1835 (4)	0.0237 (2)	0.40414 (13)	0.0291 (6)	
N2	0.5521 (4)	0.0911 (2)	0.42978 (13)	0.0286 (6)	
C1	0.3289 (4)	−0.0297 (3)	0.37562 (15)	0.0274 (7)	
C2	0.5158 (4)	0.0053 (3)	0.38798 (15)	0.0258 (7)	
C3	0.4069 (4)	0.1436 (3)	0.45887 (15)	0.0251 (7)	
C4	0.2197 (4)	0.1106 (3)	0.44487 (15)	0.0263 (7)	
C5	0.2793 (5)	−0.1318 (3)	0.33509 (17)	0.0358 (8)	
H5A	0.165563	−0.164238	0.353851	0.043*	
H5B	0.380341	−0.185522	0.340408	0.043*	
C6	0.6852 (5)	−0.0512 (3)	0.35795 (16)	0.0329 (8)	
H6A	0.651804	−0.127495	0.347866	0.039*	
H6B	0.785052	−0.052515	0.392170	0.039*	
C7	0.5692 (5)	0.0204 (3)	0.22688 (18)	0.0426 (9)	
H7A	0.477204	0.067732	0.248948	0.051*	
H7B	0.603872	0.055461	0.184170	0.051*	
C8	0.4763 (5)	−0.0915 (3)	0.21101 (18)	0.0445 (10)	
H8A	0.555159	−0.150061	0.229659	0.053*	
H8B	0.471027	−0.101211	0.161836	0.053*	
C9	0.0521 (4)	0.1650 (3)	0.47689 (16)	0.0307 (8)	
H9A	0.092472	0.197692	0.519870	0.037*	
H9B	−0.039749	0.107962	0.487395	0.037*	
C10	0.1189 (5)	0.3706 (3)	0.40868 (19)	0.0465 (10)	
H10A	0.208798	0.336051	0.378179	0.056*	
H10B	0.063285	0.432988	0.384615	0.056*	
C11	0.2260 (5)	0.4144 (3)	0.4709 (2)	0.0446 (10)	
H11A	0.162698	0.389116	0.511779	0.054*	
H11B	0.220446	0.494733	0.470435	0.054*	
C12	0.4591 (5)	0.2326 (3)	0.50841 (16)	0.0310 (8)	
H12A	0.581414	0.214425	0.528093	0.037*	
H12B	0.368141	0.231524	0.545226	0.037*	
O11	−0.1700 (4)	−0.2945 (3)	0.25969 (17)	0.0690 (9)	
O12	−0.2807 (5)	−0.3358 (3)	0.35741 (16)	0.0861 (11)	
O13	0.0187 (5)	−0.3523 (3)	0.33897 (16)	0.0757 (9)	
N10	−0.1430 (5)	−0.3274 (3)	0.31906 (19)	0.0530 (9)	

### (II) Atomic displacement parameters (Å^2^)

**Table:**

	*U*^11^	*U*^22^	*U*^33^	*U*^12^	*U*^13^	*U*^23^
Ag1	0.04418 (16)	0.03874 (17)	0.04419 (16)	0.00539 (15)	−0.00744 (11)	−0.00304 (14)
S1	0.0316 (5)	0.0397 (6)	0.0405 (5)	−0.0017 (4)	−0.0032 (4)	−0.0104 (4)
S2	0.0278 (5)	0.0356 (6)	0.0413 (5)	−0.0012 (4)	0.0058 (4)	−0.0033 (4)
S3	0.0279 (4)	0.0339 (5)	0.0399 (5)	0.0004 (4)	−0.0040 (3)	−0.0003 (4)
S4	0.0372 (5)	0.0287 (5)	0.0519 (6)	−0.0055 (4)	−0.0073 (4)	−0.0004 (4)
N1	0.0302 (15)	0.0290 (17)	0.0282 (14)	−0.0054 (13)	0.0028 (12)	−0.0022 (12)
N2	0.0258 (14)	0.0285 (17)	0.0316 (15)	0.0003 (12)	−0.0009 (11)	0.0027 (12)
C1	0.0330 (18)	0.0262 (19)	0.0231 (16)	−0.0051 (15)	0.0021 (14)	−0.0008 (14)
C2	0.0311 (17)	0.0220 (18)	0.0242 (16)	−0.0009 (15)	−0.0008 (13)	0.0013 (13)
C3	0.0309 (18)	0.0196 (18)	0.0249 (16)	−0.0026 (14)	−0.0009 (13)	0.0024 (13)
C4	0.0340 (18)	0.0227 (18)	0.0222 (16)	−0.0017 (15)	0.0016 (13)	0.0020 (13)
C5	0.040 (2)	0.028 (2)	0.040 (2)	−0.0070 (17)	0.0053 (16)	−0.0069 (15)
C6	0.0317 (18)	0.030 (2)	0.0373 (19)	0.0046 (15)	−0.0020 (15)	0.0016 (15)
C7	0.042 (2)	0.051 (3)	0.034 (2)	−0.0075 (19)	−0.0032 (16)	0.0043 (17)
C8	0.043 (2)	0.055 (3)	0.036 (2)	−0.0053 (19)	−0.0005 (16)	−0.0116 (17)
C9	0.0309 (17)	0.031 (2)	0.0305 (18)	−0.0019 (15)	0.0047 (14)	0.0017 (14)
C10	0.046 (2)	0.040 (2)	0.053 (2)	−0.0089 (19)	−0.0150 (18)	0.0132 (19)
C11	0.042 (2)	0.027 (2)	0.065 (3)	−0.0001 (17)	−0.0138 (19)	−0.0052 (18)
C12	0.0354 (18)	0.029 (2)	0.0286 (17)	−0.0012 (15)	−0.0050 (14)	−0.0029 (14)
O11	0.0629 (19)	0.068 (2)	0.077 (2)	0.0157 (18)	0.0216 (16)	0.0230 (19)
O12	0.085 (2)	0.104 (3)	0.070 (2)	−0.020 (2)	0.0425 (19)	−0.0283 (19)
O13	0.063 (2)	0.085 (3)	0.079 (2)	−0.0113 (19)	−0.0004 (17)	−0.0053 (18)
N10	0.055 (2)	0.039 (2)	0.066 (3)	−0.0064 (17)	0.021 (2)	−0.0154 (17)

### (II) Geometric parameters (Å, º)

**Table:**

Ag1—S1	2.5927 (10)	C5—H5A	0.9700
Ag1—S2^i^	2.4760 (10)	C5—H5B	0.9700
Ag1—S3^ii^	2.5382 (9)	C6—H6A	0.9700
Ag1—O11	2.492 (3)	C6—H6B	0.9700
S1—C8	1.801 (4)	C7—C8	1.533 (5)
S1—C5	1.817 (3)	C7—H7A	0.9700
S2—C7	1.809 (4)	C7—H7B	0.9700
S2—C6	1.812 (3)	C8—H8A	0.9700
S3—C10	1.805 (4)	C8—H8B	0.9700
S3—C9	1.819 (3)	C9—H9A	0.9700
S4—C11	1.806 (4)	C9—H9B	0.9700
S4—C12	1.807 (3)	C10—C11	1.524 (5)
N1—C1	1.340 (4)	C10—H10A	0.9700
N1—C4	1.341 (4)	C10—H10B	0.9700
N2—C3	1.338 (4)	C11—H11A	0.9700
N2—C2	1.343 (4)	C11—H11B	0.9700
C1—C2	1.408 (4)	C12—H12A	0.9700
C1—C5	1.505 (4)	C12—H12B	0.9700
C2—C6	1.503 (4)	O11—N10	1.241 (4)
C3—C4	1.409 (4)	O12—N10	1.239 (4)
C3—C12	1.491 (4)	O13—N10	1.243 (4)
C4—C9	1.498 (4)		
			
S2^i^—Ag1—S1	132.51 (3)	H6A—C6—H6B	107.5
S3^ii^—Ag1—S1	97.47 (3)	C8—C7—S2	114.4 (3)
S2^i^—Ag1—S3^ii^	121.65 (3)	C8—C7—H7A	108.7
O11—Ag1—S1	93.62 (8)	S2—C7—H7A	108.7
S2^i^—Ag1—O11	97.12 (8)	C8—C7—H7B	108.7
O11—Ag1—S3^ii^	109.25 (8)	S2—C7—H7B	108.7
C8—S1—C5	104.04 (16)	H7A—C7—H7B	107.6
C8—S1—Ag1	103.02 (12)	C7—C8—S1	114.1 (3)
C5—S1—Ag1	105.21 (11)	C7—C8—H8A	108.7
C7—S2—C6	102.43 (16)	S1—C8—H8A	108.7
C7—S2—Ag1^iii^	105.67 (13)	C7—C8—H8B	108.7
C6—S2—Ag1^iii^	109.21 (12)	S1—C8—H8B	108.7
C10—S3—C9	104.08 (16)	H8A—C8—H8B	107.6
C10—S3—Ag1^iv^	98.75 (13)	C4—C9—S3	116.5 (2)
C9—S3—Ag1^iv^	111.15 (11)	C4—C9—H9A	108.2
C11—S4—C12	103.52 (17)	S3—C9—H9A	108.2
C1—N1—C4	118.7 (3)	C4—C9—H9B	108.2
C3—N2—C2	118.7 (3)	S3—C9—H9B	108.2
N1—C1—C2	120.5 (3)	H9A—C9—H9B	107.3
N1—C1—C5	115.9 (3)	C11—C10—S3	115.5 (3)
C2—C1—C5	123.5 (3)	C11—C10—H10A	108.4
N2—C2—C1	120.7 (3)	S3—C10—H10A	108.4
N2—C2—C6	116.0 (3)	C11—C10—H10B	108.4
C1—C2—C6	123.2 (3)	S3—C10—H10B	108.4
N2—C3—C4	120.5 (3)	H10A—C10—H10B	107.5
N2—C3—C12	115.5 (3)	C10—C11—S4	114.3 (3)
C4—C3—C12	123.9 (3)	C10—C11—H11A	108.7
N1—C4—C3	120.8 (3)	S4—C11—H11A	108.7
N1—C4—C9	116.3 (3)	C10—C11—H11B	108.7
C3—C4—C9	122.8 (3)	S4—C11—H11B	108.7
C1—C5—S1	114.0 (2)	H11A—C11—H11B	107.6
C1—C5—H5A	108.7	C3—C12—S4	116.8 (2)
S1—C5—H5A	108.7	C3—C12—H12A	108.1
C1—C5—H5B	108.7	S4—C12—H12A	108.1
S1—C5—H5B	108.7	C3—C12—H12B	108.1
H5A—C5—H5B	107.6	S4—C12—H12B	108.1
C2—C6—S2	115.3 (2)	H12A—C12—H12B	107.3
C2—C6—H6A	108.4	N10—O11—Ag1	110.8 (2)
S2—C6—H6A	108.4	O12—N10—O11	118.5 (4)
C2—C6—H6B	108.4	O12—N10—O13	121.1 (4)
S2—C6—H6B	108.4	O11—N10—O13	120.4 (3)
			
C4—N1—C1—C2	−0.4 (4)	C1—C2—C6—S2	97.0 (3)
C4—N1—C1—C5	175.3 (3)	C7—S2—C6—C2	−52.5 (3)
C3—N2—C2—C1	−0.8 (4)	Ag1^iii^—S2—C6—C2	59.2 (3)
C3—N2—C2—C6	−179.0 (3)	C6—S2—C7—C8	−60.0 (3)
N1—C1—C2—N2	1.6 (5)	Ag1^iii^—S2—C7—C8	−174.3 (2)
C5—C1—C2—N2	−173.7 (3)	S2—C7—C8—S1	115.8 (3)
N1—C1—C2—C6	179.7 (3)	C5—S1—C8—C7	−76.5 (3)
C5—C1—C2—C6	4.3 (5)	Ag1—S1—C8—C7	173.9 (2)
C2—N2—C3—C4	−1.1 (4)	N1—C4—C9—S3	−86.5 (3)
C2—N2—C3—C12	175.8 (3)	C3—C4—C9—S3	96.9 (3)
C1—N1—C4—C3	−1.6 (4)	C10—S3—C9—C4	−56.6 (3)
C1—N1—C4—C9	−178.3 (3)	Ag1^iv^—S3—C9—C4	48.7 (3)
N2—C3—C4—N1	2.4 (4)	C9—S3—C10—C11	−54.1 (3)
C12—C3—C4—N1	−174.3 (3)	Ag1^iv^—S3—C10—C11	−168.6 (3)
N2—C3—C4—C9	178.9 (3)	S3—C10—C11—S4	113.1 (3)
C12—C3—C4—C9	2.2 (5)	C12—S4—C11—C10	−79.7 (3)
N1—C1—C5—S1	92.7 (3)	N2—C3—C12—S4	94.2 (3)
C2—C1—C5—S1	−91.7 (3)	C4—C3—C12—S4	−89.0 (3)
C8—S1—C5—C1	77.0 (3)	C11—S4—C12—C3	78.9 (3)
Ag1—S1—C5—C1	−175.0 (2)	Ag1—O11—N10—O12	−177.6 (3)
N2—C2—C6—S2	−84.9 (3)	Ag1—O11—N10—O13	1.7 (4)

Symmetry codes: (i) −*x*+1, *y*−1/2, −*z*+1/2; (ii) −*x*, *y*−1/2, −*z*+1/2; (iii) −*x*+1, *y*+1/2, −*z*+1/2; (iv) −*x*, *y*+1/2, −*z*+1/2.

### (II) Hydrogen-bond geometry (Å, º)

**Table:**

*D*—H···*A*	*D*—H	H···*A*	*D*···*A*	*D*—H···*A*
C5—H5*A*···O13	0.97	2.51	3.239 (5)	132
C6—H6*A*···O12^v^	0.97	2.56	3.442 (5)	150
C8—H8*B*···S4^i^	0.97	2.74	3.696 (4)	169
C11—H11*B*···S4^vi^	0.97	2.91	3.508 (4)	121
C12—H12*B*···O12^vii^	0.97	2.37	3.177 (4)	140

Symmetry codes: (i) −*x*+1, *y*−1/2, −*z*+1/2; (v) *x*+1, *y*, *z*; (vi) −*x*+1, −*y*+1, −*z*+1; (vii) −*x*, −*y*, −*z*+1.
